# Spheroids reveal hypoxia‑driven spatial restriction of adenoviral infection

**DOI:** 10.1038/s41598-026-53319-4

**Published:** 2026-05-21

**Authors:** Tamara Büttner, Xiaoyan Wang, Brenda Krishnacoumar, Athanasios Papadamakis, Suna Cicek, Yves Schild, Sandra Winning, Elisabeth Littwitz-Salomon, Anja Ehrhardt, Ulf Dittmer, Wibke Bayer, Joachim Fandrey, Anna Malyshkina

**Affiliations:** 1 Institute of Physiology, University of Duisburg-Essen, University Hospital Essen, Essen, Germany; 2 Institute for Virology, University of Duisburg-Essen, University Hospital Essen, Essen, Germany; 3 Virology and Microbiology, Center for Biomedical Education and Research (ZBAF), Faculty of Health, Witten/Herdecke University, Witten, Germany; 4 Institute for the Research on HIV and AIDS-associated Diseases, University of Duisburg-Essen, University Hospital Essen, Essen, Germany

**Keywords:** Cancer, Microbiology, Oncology

## Abstract

Hypoxia is a hallmark of solid tumors and represents a major barrier for effective cancer therapies, including oncolytic virotherapy. While adenoviruses are widely studied as oncolytic agents, the impact of tumor-associated hypoxia on viral infection and spatial spread remains incompletely understood. Here, we investigated how oxygen availability influences adenovirus infection in two-dimensional (2D) cultures and three-dimensional (3D) tumor spheroids. We confirmed that cell lines commonly used in adenovirus research (HEK293A, A549), as well as KP4 pancreatic cancer cells, exhibited a physiological response to hypoxia. In KP4 monolayers, hypoxia strongly reduced adenoviral protein production. To model oxygen gradients found in solid tumors, we established stable KP4 spheroids and performed spatial analysis of HAdV5_GFP infection. When virus was added during spheroid formation and hypoxia development, infection was largely restricted to the well-oxygenated outer rim. In contrast, inoculation of virus under normoxia prior to spheroid formation resulted in a more uniform distribution of infected cells throughout the spheroid. Together, our findings demonstrate that hypoxia not only suppresses adenoviral replication in cell culture but also shapes the spatial pattern of infection in 3D tumor models, highlighting the importance of hypoxia-relevant 3D systems in preclinical evaluation of oncolytic adenoviruses.

**Supplementary Information:**

The online version contains supplementary material available at 10.1038/s41598-026-53319-4.

## Introduction

Oncolytic viruses (OV) represent a promising class of emerging anticancer immunotherapies, utilizing the natural ability of certain replication-competent viruses to selectively infect and destroy tumor cells while sparing normal, non-neoplastic tissues. Oncolytic human adenoviruses (HAdV), in particular, are a prominent focus in the development of cancer therapies. First, they have a HAdV type-specific natural tropism for a broad variety of cell types, allowing efficient tumor cell targeting. Second, their genome can accommodate large inserts, making them highly versatile for therapeutic engineering. Additionally, adenoviruses do not integrate into the host genome and stimulate strong immune response, amplifying antitumor activity^1^.

Effective cancer treatment using oncolytic HAdV relies on the viral spread from infected to uninfected cells. To date, there has been limited success in clinical studies in the use of replicating adenoviruses. For instance, Tasadenoturev, HAdV type 5-based OV, showed a strong safety profile but achieved only modest efficacy in both pediatric^2^ and adult^3^ glioma patients. Along these lines, CG0070, a GM-CSF-armed HAdV type 5-based oncolytic virus developed for bladder cancer, demonstrated mixed results and did not become a widely adoptive option^4^. Similarly, Oncorine, another HAdV5-based therapy approved in China, elicited improved responses in certain settings, yet its broader clinical use remains limited^5,6^. While these examples do not achieve complete therapeutic efficacy, they highlight the potential of oncolytic adenoviruses as a promising approach that requires further investigation.

The difference between laboratory findings and clinical results may arise from multiple factors, including preexisting immunity against the administered virus^7^, the dense extracellular matrix and aberrant tumor vasculature that generate interstitial hypertension^8^, and the negative impact of tumor hypoxia. Hypoxia, the condition characterized by low oxygen levels, is frequently observed in solid tumors^9,10^. It is difficult to determine the hypoxic state in tumors due to variations in oxygen content between tissues, as well as differences in tumor size and measurement methods, and tissue oxygenation is highly variable, also within the same organ. However, the available results indicate that the measurement of tumor partial pressure of oxygen (pO_2_) in patients (polarographic technique) has demonstrated the presence of low values (< 10 mmHg) or equal 1,3% O_2_ in several different tumor types, including pancreatic cancer, head and neck tumors, breast cancer, cervical cancer, and melanoma. Specifically, the tumor microenvironment exhibits a median O₂ concentration of 1.3%^11,12^. In contrast, adenoviruses naturally infect tissues that are typically exposed to normal ambient oxygen concentrations (normoxia). Moreover, it was shown that hypoxia reduces adenoviral replication in 2D cell culture^13,14^.

Sustained interest in the development of 3D culture models in recent years reflects the growing demand for physiologically relevant systems in cancer research. These models are particularly valuable in tumor biology, as they recapitulate key features of the tumor microenvironment, including oxygen gradients. Spheroids typically display proliferative, normoxic (or more precisely: physoxic) cells at the periphery, an intermediate hypoxic zone, and a central region that is often severely hypoxic, anoxic, and/or necrotic. Importantly, such gradients arise intrinsically during spheroid formation, avoiding the need for external hypoxic chambers. As a result, spheroids provide a more natural representation of tumor physiology, hypoxia-driven processes, and therapeutic responses. Thus, they are suitable to enhance the predictive value of preclinical studies for in vivo and clinical outcomes.

In the present study, we examined how the intrinsic oxygen gradients that develop within spheroids influence HAdV infection. Our results show the hypoxic regions in the inner part of the spheroid which strongly correlate with limited infection rates. Thus, we indicate that such spheroids represent a valuable tool for cancer research and virotherapy. Specifically, we determined that 3D spheroid cultures recapitulate the inhibitory effects of hypoxia observed in 2D monolayers, thereby providing a more physiologically relevant model to study adenovirus replication in the context of tumor biology.

## Results

### Hypoxia stabilizes HIF-1α in A549, HEK293A, and KP4 cells

**Fig. 1:**
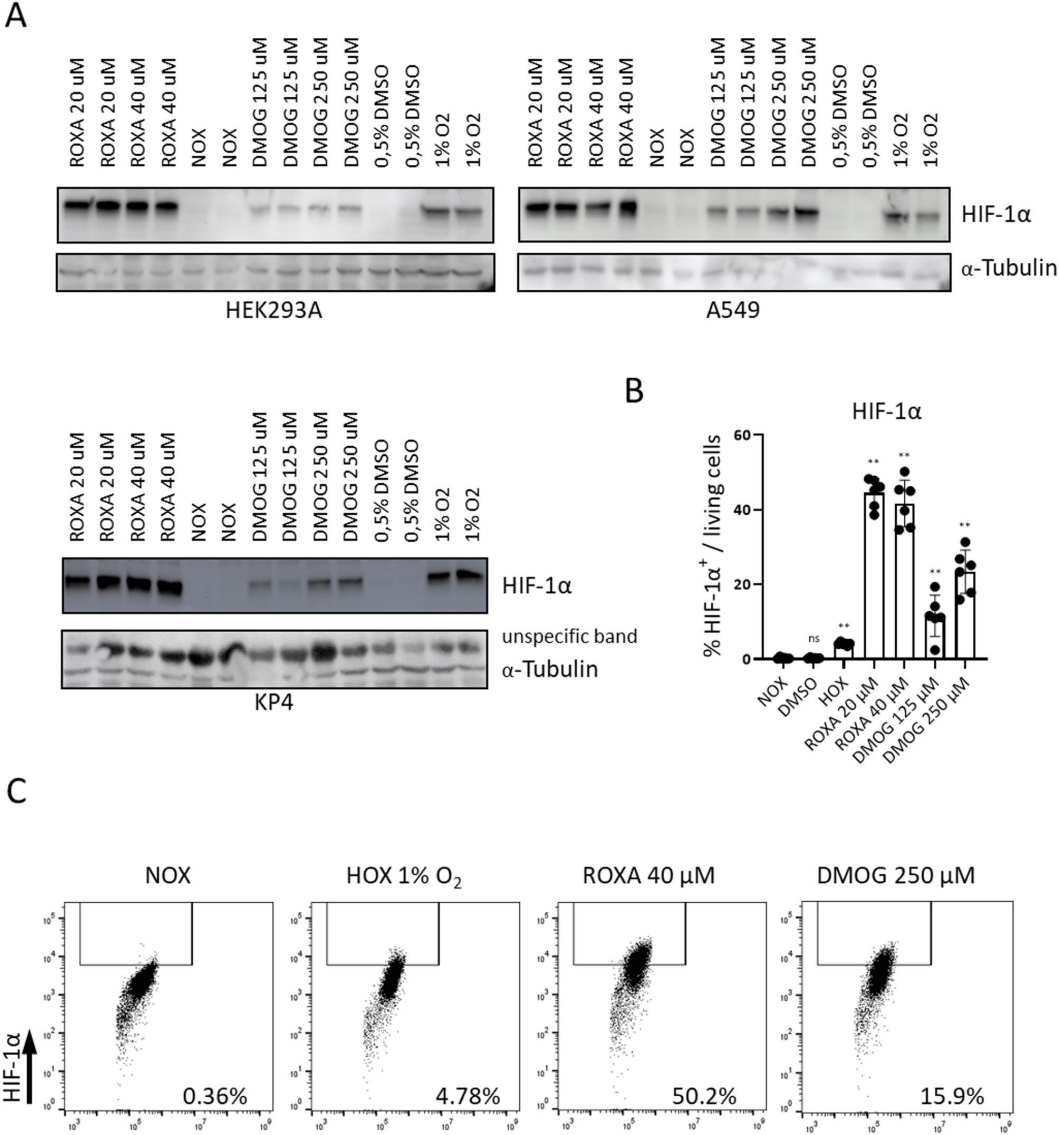
HIF-1α levels are stabilized under hypoxia and after treatment with prolyl hydroxylase (PHD) inhibitors. (**A**) HEK293A, A549 and KP4 cells were seeded in monolayer and cultured for 4 h under normoxia (NOX) or hypoxia (HOX, 1% O₂), or treated under normoxia with the PHD inhibitors Roxadustat (ROXA; 20 or 40 µM) or Dimethyloxalylglycine (DMOG; 125 or 250 µM), with 0.5% DMSO as vehicle control. Whole‑cell lysates were analyzed by Western blotting for HIF‑1α; α‑tubulin or an unspecific band served as loading controls. Cropped regions of blots are shown. The original uncropped blots are presented in Supplementary Figure 1. (**B**) KP4 cells were cultured for 4 h under normoxia (NOX), hypoxia (HOX, 1% O₂), 0.5% DMSO, ROXA (20 or 40 µM) or DMOG (125 or 250 µM), stained intracellularly for HIF‑1α and analyzed by flow cytometry. The graph shows the percentage of HIF‑1α⁺ cells among viable cells. Symbols represent individual samples (n = 6) and bars indicate mean ± SD. Shown results of two independent experiments. Each group was compared to NOX group using Mann-Whitney test. ns, not significant; ** p < 0,05. (**C**) Representative flow cytometry dot plots of HIF‑1α staining in KP4 cells cultured under normoxia (NOX), hypoxia (HOX, 1% O₂), ROXA 40 µM or DMOG 250 µM, with the HIF‑1α⁺ gate indicated and the corresponding percentages shown.

First, we selected human cell lines as model systems: A549 lung carcinoma and HEK293A human embryonic kidney cells, which are widely used in adenovirus research^15^, and KP4 pancreatic cancer cells, a line commonly used in tumor studies^16^. Moreover, all three models formed stable spheroids, providing a suitable system to study hypoxic conditions in a three-dimensional context^17–19^. To verify that these cell lines mount a physiological response to hypoxia, we exposed them to 1% O₂ and analyzed HIF-1α stabilization via Western blot. Cells were cultured for four hours either under ambient oxygen or hypoxic conditions (1% O₂). In parallel, cells were treated with the prolyl hydroxylase (PHD) inhibitors Roxadustat or Dimethyloxalylglycine (DMOG) at various concentrations, serving as positive controls for HIF-1α stabilization. Hypoxia and PHD inhibition both led to a clear accumulation of HIF-1α protein in all three cell lines (Fig. 1A).​

To further validate HIF-1α induction in KP4 cells, the experiment was repeated and HIF-1α levels were quantified by flow cytometry. Pharmacologic PHD inhibition with Roxadustat (20 or 40 µM) increased the proportion of HIF-1α⁺ KP4 cells to ~ 45% and ~ 42%, respectively, whereas DMOG (125 or 250 µM) increased it to ~ 12% and ~ 23%. Hypoxia alone elevated this population to approximately 4%. (Fig. 1B, C). Together, these data demonstrate that the chosen cell models retain a hypoxia-sensing pathway and respond to low oxygen tension or PHD inhibition with clear HIF-1α stabilization.

### All selected cell lines form spheroids, but only KP4 cells generate homogeneous spheroids suitable for sectioning

**Fig. 2:**
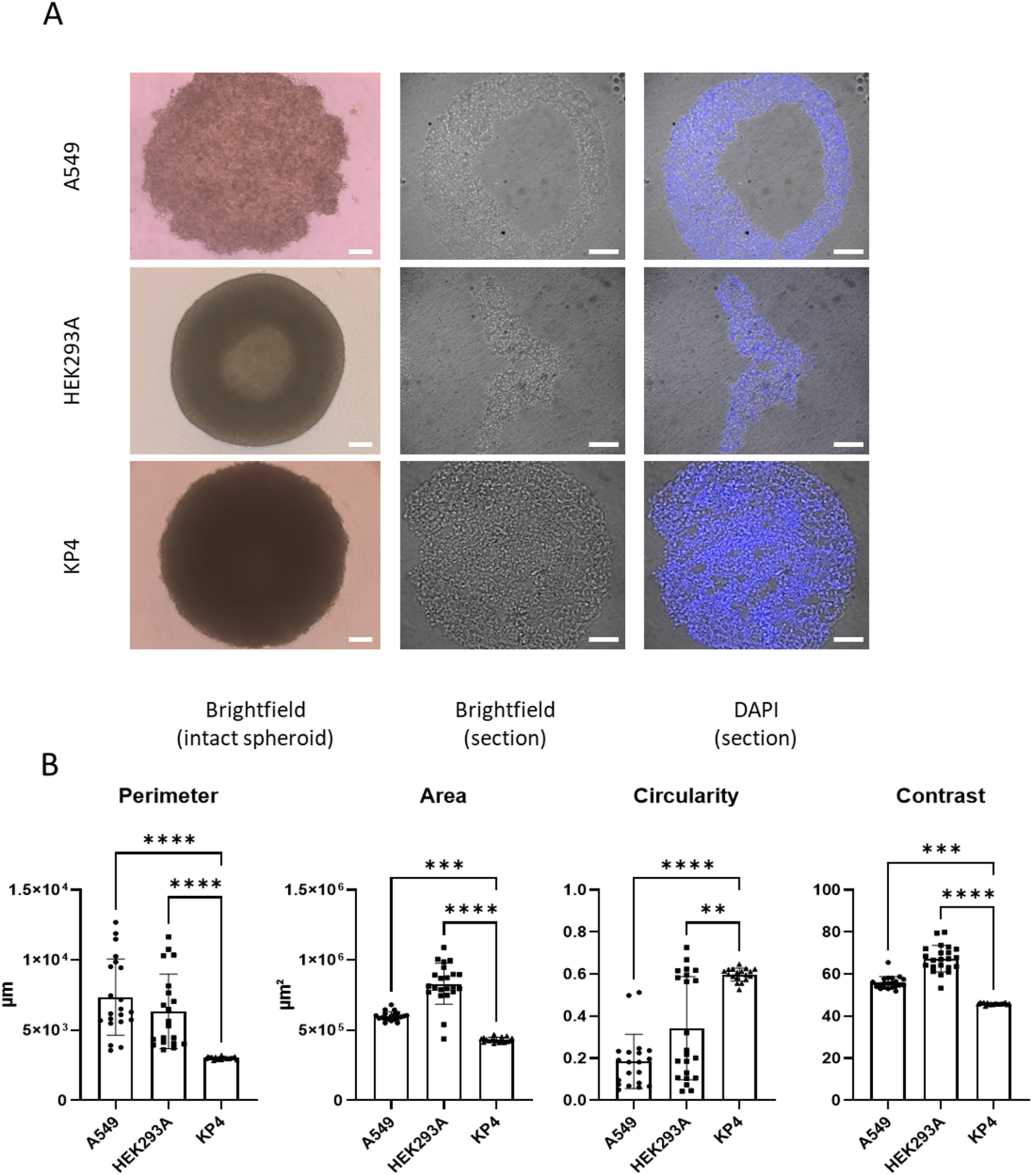
KP4 cells form compact, homogeneous spheroids suitable for sectioning. (**A**) Representative spheroids generated from A549, HEK293A and KP4 cells using the hanging‑drop method. Left column: brightfield overview images of intact spheroids. Middle column: brightfield images of spheroid sections. Right column: corresponding sections stained with DAPI to visualize nuclei. Scale bars, 100 µm. (**B**) Quantitative analysis of spheroid morphology. Perimeter, area, circularity and contrast were measured after 5 days of hanging‑drop culture from brightfield images using the Insidia macro software (Fiji/ImageJ). Circularity is reported as a dimensionless index ranging from 0 to 1, where 1 indicates a perfect circular shape. Contrast values represent a unitless grayscale heterogeneity index, with higher values indicating greater internal intensity variation within the spheroid and does not represent absolute fluorescence units. Data points represent individual spheroids (n = 18–22 spheroids per cell line); bars indicate mean ± SD. Statistical significance was assessed using one‑way ANOVA (**** p < 0.0001, *** p < 0.001,** p < 0.01).

Because subsequent analyses required intact tissue sections, we first identified cell lines capable of forming spheroids suitable for histological processing. A549, HEK293A and KP4 cells were cultured for 5 days using the hanging-drop method and examined by microscopy. Brightfield images of intact aggregates confirmed that all three cell lines were able to form compact spheroids. Microscopic analysis of sections, however, revealed marked differences in architecture between the models (Fig. 2 A). A549 formed spheroids with loose or irregular borders, while HEK293A spheroids displayed a heterogeneous cell distribution. In contrast, KP4 cells reproducibly generated dense, spherical structures with sharp edges and uniform cellular architecture.

When spheroids were snap-frozen and sectioned, these structural differences translated into distinct handling properties. A549 and HEK293A spheroids were fragile and were frequently distorted or disrupted during cutting, while KP4 spheroids largely retained their integrity and could be reliably sectioned. To quantify these observations, spheroid morphology was analyzed using the Insidia image-analysis macro. KP4 spheroids displayed significantly smaller perimeter, area, and lower image contrast, but higher circularity compared with A549 and HEK293A aggregates (Fig. 2B). These metrics indicate that KP4 spheroids are more compact and homogeneous, and therefore represent the most suitable model for downstream histological sectioning and subsequent experiments.

### There is a hypoxic gradient in spheroids

Next, we aimed to determine whether a hypoxic microenvironment develops within the spheroid core, as described in solid tumors. To this end, the hypoxia-sensitive fluorescent dye Hypoxia Green was added during spheroid formation. This probe exhibits low basal fluorescence even in normoxic cells but undergoes enzymatic reduction under low oxygen, resulting in the release of a fluorophore that produces increased green fluorescence in hypoxic cells.

**Fig. 3:**
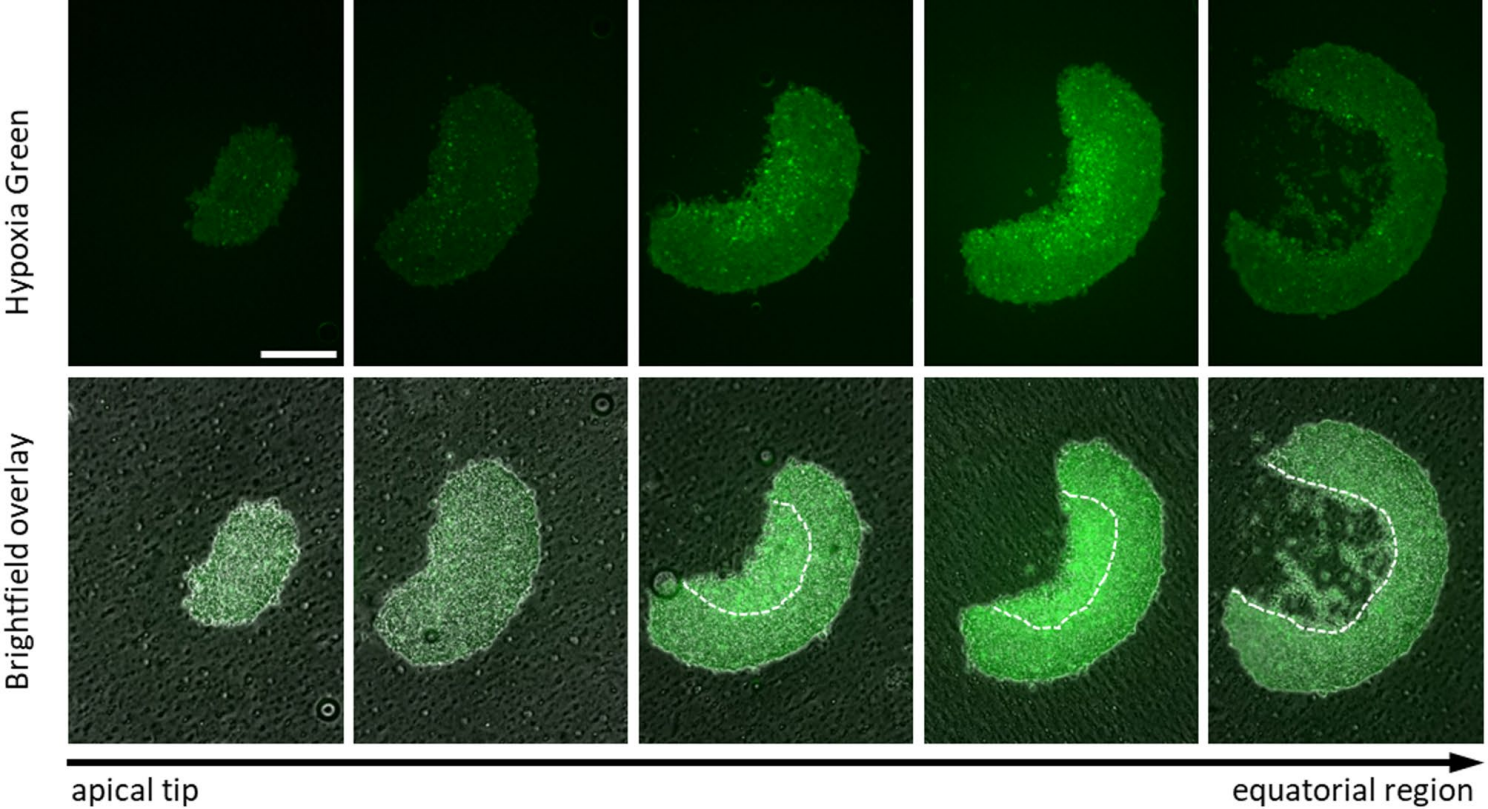
Three‑dimensional mapping of hypoxic regions in KP4 spheroid. Cryosections of KP4 spheroids formed in the presence of the hypoxia‑sensitive Green Hypoxia reagent were imaged along the apical–basal axis from the apical tip (left) toward the widest equatorial region (right). The upper row shows fluorescence images (Hypoxia Green signal), and the lower row shows corresponding fluorescence overlays on brightfield images. White dashed lines indicate the inner hypoxic zone. Scale bar, 100 μm.

Following spheroid formation, samples were fixed and cryosectioned across the entire spheroid thickness to enable microscopic assessment of the spatial distribution of hypoxic regions. Figure 3 shows representative images of consecutive cryosections arranged from the apical tip of the spheroid toward its widest equatorial region. The upper row displays the green fluorescence channel alone to facilitate detection of hypoxic areas, whereas the lower row shows overlays of green fluorescence with brightfield images to visualize spheroid morphology. Across all sections, a diffuse basal fluorescence signal was detected throughout the spheroid structure. In the first two sections derived from the apical region, no areas of markedly increased fluorescence were observed. In contrast, in the two subsequent sections obtained from a broader region above the spheroid midpoint, clusters of brightly fluorescent cells were detected and were localized along the inner concave surface. The section corresponding to the widest region of the spheroid exhibited a ring-shaped morphology with partial structural disruption due to loss of tissue integrity during sectioning, indicating a necrotic and unstable core. This observation is consistent with a hypoxic gradient commonly described in solid tumors, with peripheral normoxic cells and a hypoxic zone surrounding a central anoxic and/or necrotic core.

### KP4 cell line supports efficient adenoviral infection

**Fig. 4:**
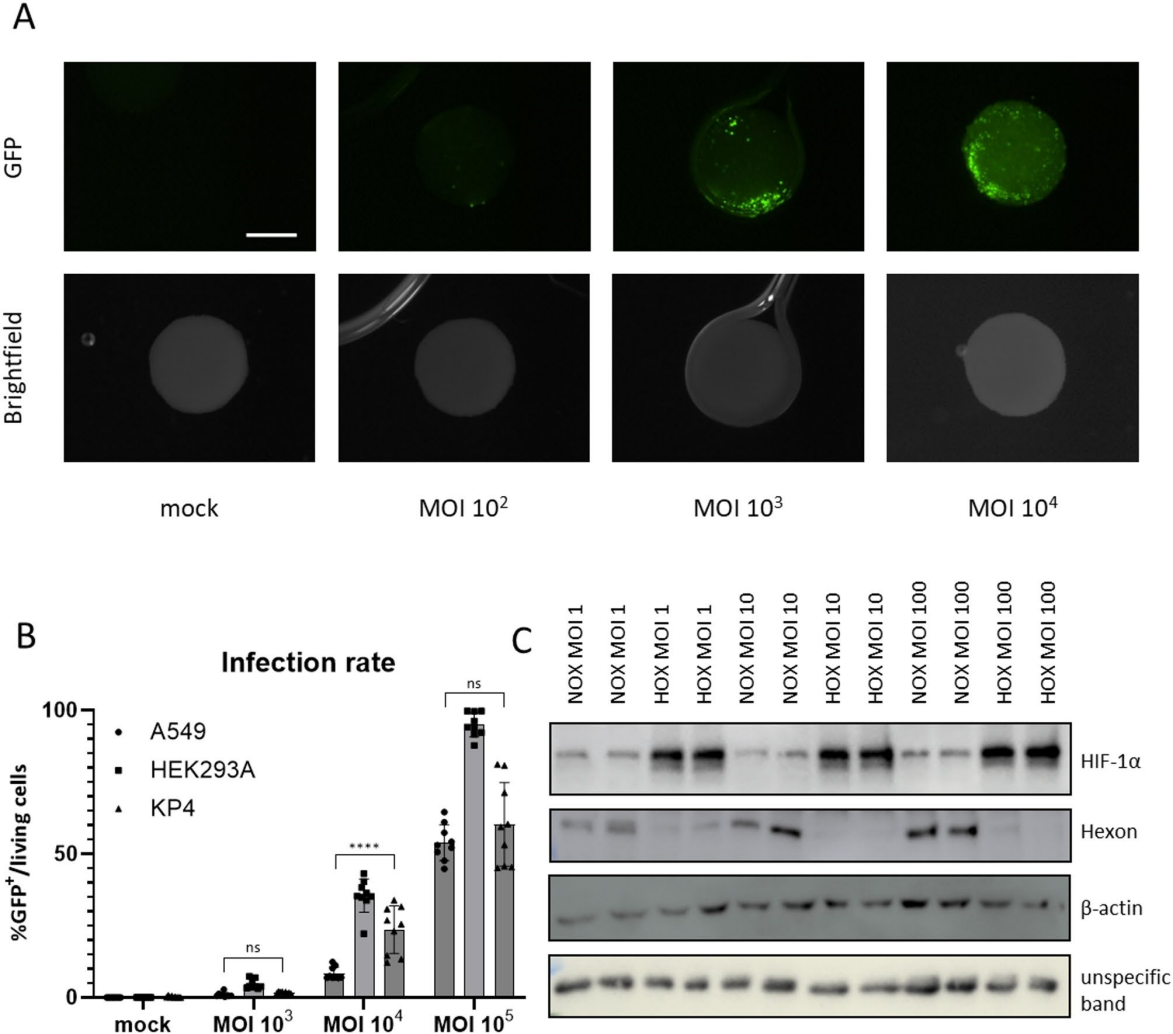
KP4 cells are permissive to HAdV5 infection and show hypoxia-dependent reduction of viral protein expression. (**A**) KP4 cells were infected in single-cell suspension with increasing multiplicities of infection (MOI) of HAdV5_GFP or left uninfected (mock) and immediately seeded as hanging-drop cultures for spheroid formation. Spheroids were collected 5 days later, fixed in 4% paraformaldehyde and imaged by fluorescence (GFP) and brightfield microscopy. Representative *en face* views are shown. The scale bar represents 100 μm. (**B**) Monolayer cultures of HEK293A, A549 and KP4 cells were infected with the indicated MOI of HAdV5_GFP or left uninfected (mock). At 24 h post infection, cells were harvested, stained for viability and analyzed by flow cytometry. The percentage of GFP⁺ viable cells was taken as the infection rate. Bars represent mean ± SD of biological replicates. Shown results of three independent experiments. For each MOI, infection rates were compared using Mann-Whitney test. ns, not significant; **** p < 0,0001. (**C**) KP4 cells were infected under ambient oxygen for 2 h with the indicated MOI of HAdV5 and cultured for 24 h under normoxia or hypoxia (1% O₂) before lysis and immunoblotting. Representative blots for HIF-1α and Hexon are shown, together with an unspecific band and β-actin used as a loading control. Samples derive from the same experiment and the blots were processed in parallel. Cropped regions of blots are shown. The original uncropped blots are presented in Supplementary Fig. 1.

Because KP4 cells showed the most reproducible spheroid formation and the desired hypoxic gradient, they were selected for subsequent experiments. HAdV5 entry depends largely on Coxsackie and Adenovirus Receptor (CAR), which is highly expressed in model lines such as HEK293 derivatives and A549 cells, contributing to their wide use in adenovirus research. In contrast, several pancreatic cancer cell lines have been reported to downregulate CAR at the cell surface^20^, raising concerns about their permissiveness to HAdV5. Therefore, to test infectability in the spheroid context, single-cell suspensions of KP4 cells were infected with escalating multiplicities of infection (MOI) of HAdV5_GFP or left uninfected and immediately transferred into hanging drops to allow spheroid formation (Fig. 4 A). Five days later, spheroids were collected and analyzed for GFP expression. KP4 spheroids displayed a detectable GFP signal already at the lowest tested MOI and showed a clear increase in GFP intensity with higher viral doses, indicating efficient adenoviral infection.

To benchmark KP4 cells against established adenoviral target cell lines, 2D cultures of HEK293A, A549, and KP4 cells were infected with increasing MOIs of HAdV5_GFP or left uninfected and analyzed 24 h later by flow cytometry for the percentage of GFP⁺ viable cells (Fig. 4B). As expected, HEK293A cells showed the highest proportion of infected cells across conditions. Notably, KP4 cells consistently reached infection rates comparable to, and at 10^4^ MOI even exceeding, those observed in A549 cells, supporting KP4 as a suitable and permissive model for adenoviral infection.

### KP4 cell line shows reduced viral protein production under hypoxia

Next, the effect of hypoxia on viral protein production in KP4 cells was examined, given previous reports that adenoviral gene expression is strongly reduced under low oxygen tension in several tumor cell types, including lung (A549, H1299), prostate (LNCaP), and colorectal cancer cells (HT29, DLD-1)^13,14^. KP4 monolayers were infected under ambient oxygen for 2 h with increasing MOIs of HAdV5, then cultured either under ambient oxygen or 1% O₂ for 24 h before protein extraction and Western blot analysis. Under hypoxia, HIF-1α was stabilized as expected, confirming an effective hypoxic response. As a representative adenoviral structural marker, we analyzed Hexon, the major capsid protein of HAdV5. Hexon protein levels were markedly diminished across all tested MOIs, whereas β‑actin remained unchanged (Fig. 4 C). This indicates inhibition at the level of viral protein synthesis or late gene expression, rather than merely impaired viral entry. Therefore, similar to other tumor cell lines, KP4 cells exhibit strong hypoxia-mediated suppression of adenoviral protein production.

### HAdV5 infection is restricted to the periphery in KP4 spheroids

**Fig. 5:**
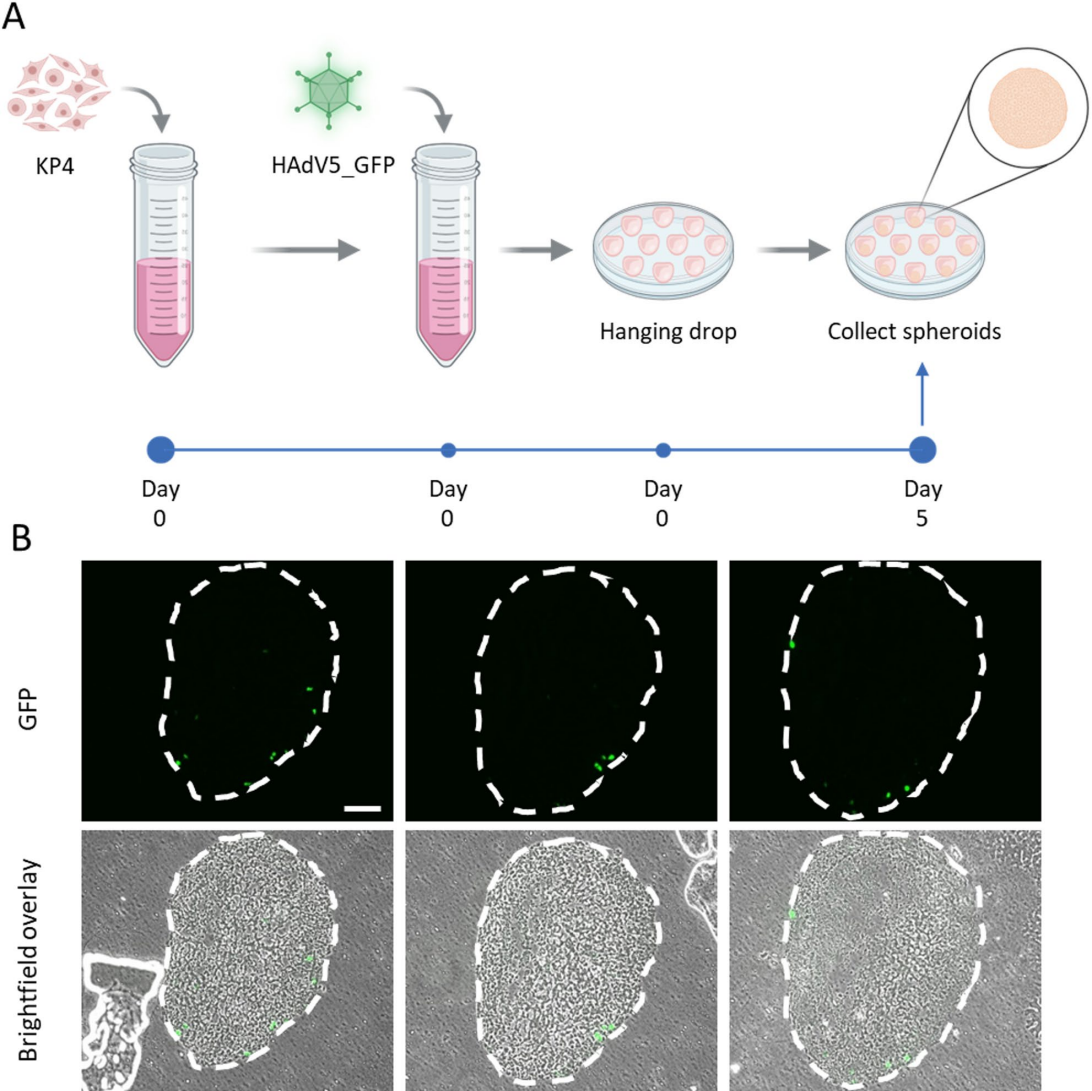
KP4 spheroids show peripheral localization of HAdV5-infected cells. (**A**) Schematic of the experimental workflow. KP4 cells were infected with fluorescently labelled HAdV5_GFP at MOI 1000 on day 0, immediately transferred to hanging‑drop culture to form spheroids, and maintained for 5 days before collection and fixation. Created with Biorender.com. (**B**) Serial cryosections from one representative spheroid show GFP fluorescence (top row) and corresponding brightfield–GFP overlays (bottom row). Dashed white lines outline the spheroid boundary. Scale bar, 100 μm. See also Fig. 6 C for quantitative analysis.

Our next step was to investigate the spatial distribution of infected cells within KP4 spheroids. Since hypoxia strongly reduced adenoviral protein production in KP4 monolayers and spheroids contain a hypoxic core, we asked ourselves whether infection would be restricted to the better-oxygenated outer layers. To test this, KP4 cells were infected with fluorescently labelled HAdV5_GFP and immediately transferred to hanging‑drop culture so that viral infection and spheroid formation occurred in parallel. Mature spheroids were harvested and fixed 5 days later (Fig. 5 A).

First, we assessed the fixed spheroids by z-stack fluorescence microscopy. It showed that in the vast majority of cases HAdV5_GFP^+^ cells were restricted to the spheroid surface, with little or no GFP signal detectable in the central regions (Supplementary Video 1). Infected cells localized almost exclusively to the peripheral layers, whereas the spheroid cores remained largely GFP‑negative, indicating a strong bias of infection toward the outer normoxic zones. However, the approach of z-stack microscopy is limited by incomplete optical penetration through the full spheroid thickness and by out‑of‑focus fluorescence from neighboring planes, which can complicate precise assignment of signal to specific depths.

To overcome these limitations, spheroids were frozen, cryosectioned and analyzed on serial sections centered around the maximal diameter. In these physical sections, out‑of‑plane fluorescence was eliminated and the entire cross‑sectional area could be evaluated. Consistent with the z‑stack analysis, HAdV5_GFP^+^ cells were observed almost exclusively at the spheroid periphery (Fig. 5B). These findings indicate that the adenoviral infection within KP4 spheroids is restricted to the better oxygenated outer layers.

### Normoxic infection before spheroid formation abolishes peripheral restriction of HAdV5 in KP4 spheroids

**Fig. 6:**
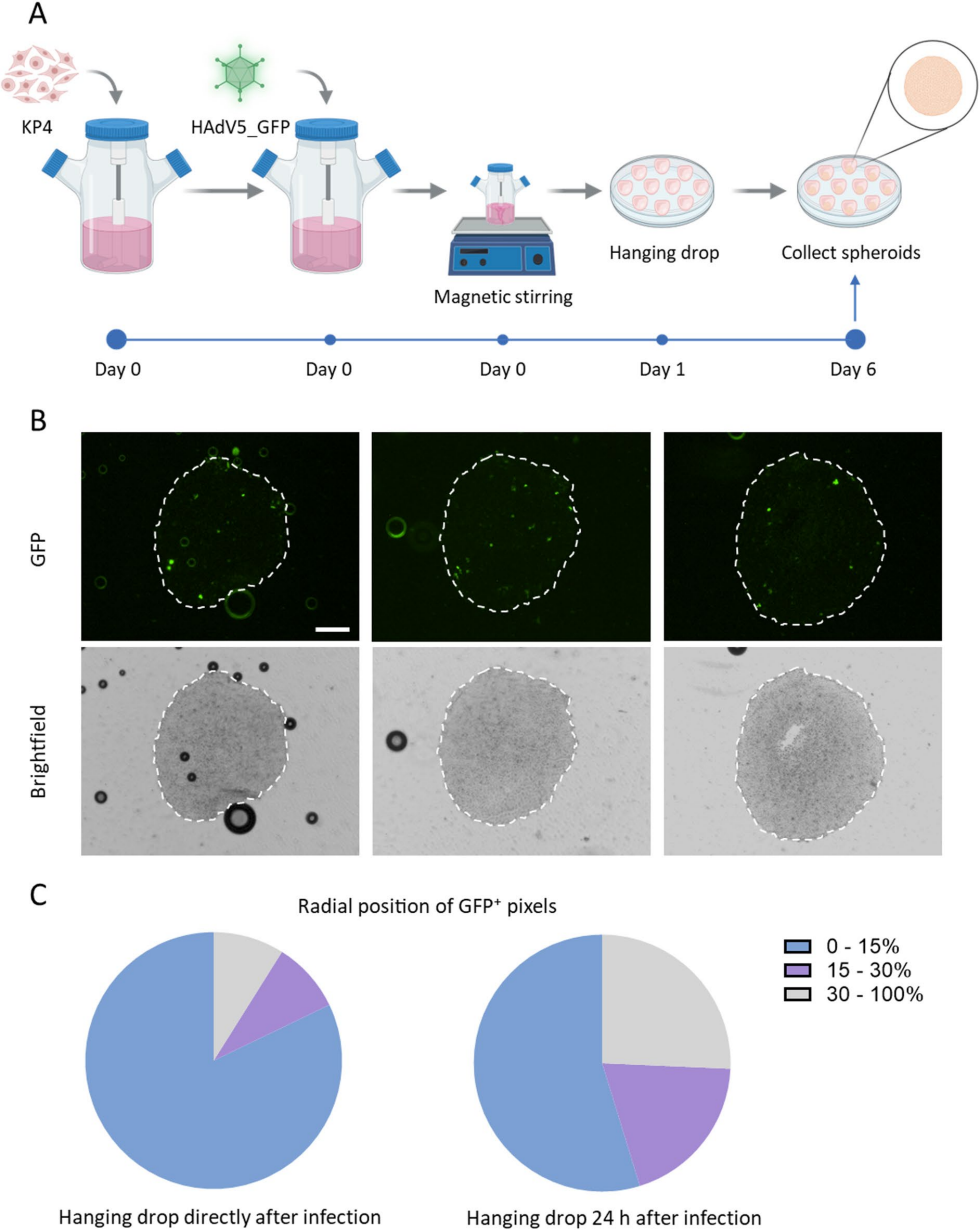
Infection prior to spheroid formation results in uniform distribution of infected cells within KP4 spheroids. (**A**) Schematic of the experimental workflow. KP4 cells were infected with fluorescently labelled HAdV5_GFP at MOI 1000 on day 0 and maintained for 24 h under magnetic stirring in specialized culture flask. The infected cell suspension was then transferred to hanging‑drop culture to allow spheroid formation, and spheroids were collected and fixed on day 6 post infection. Created with Biorender.com. (**B**) Representative cryosections of three independent spheroids show GFP fluorescence (top row) and the corresponding brightfield images (bottom row). Dashed white lines outline the spheroid boundary. Scale bar, 200 μm. **(C)** Quantitative analysis of the radial position of GFP⁺ pixels. Distances from the spheroid surface were binned into three concentric regions: surface to 15% of spheroid radius (0 ≤ value < 15), 15 to 30% of spheroid radius (15 ≤ value < 30), and inner core (30 ≤ value ≤ 100). Pie charts illustrate the overall proportion of HAdV5-infected cells falling into each region (n = 8–10 sections per condition).

Spheroids formed immediately after infection restricted HAdV5_GFP‑positive cells to the spheroid periphery. Here, the establishment of the hypoxic gradient occurred in parallel with viral infection. To test whether this peripheral restriction could be relieved when infection takes place entirely under normoxic conditions, KP4 cells were infected with HAdV5_GFP in single‑cell suspension and maintained for 24 h on a magnetic stirrer in specialized culture flasks to ensure continuous mixing. After this infection period, cells were transferred to hanging‑drop culture to form spheroids, which were harvested and fixed 5 days later (Fig. 6 A).

As before, spheroids were first analyzed by z‑stack fluorescence microscopy. In contrast to spheroids placed to hanging drops immediately after infection, these samples no longer showed an obviously peripheral pattern of HAdV5_GFP^+^ cells. Instead, infection appeared more uniformly distributed throughout the spheroid volume (Supplementary Video 2). Consistently, cryosections of frozen spheroids revealed GFP^+^ cells not only at the outer rim but also deeper within the spheroid mass, indicating a more heterogeneous distribution than in spheroids infected during hypoxia gradient formation (Fig. 6B).

To quantitatively compare the spatial distribution of infected cells between the two conditions, the spheroid radius was divided into concentric shells corresponding to 0–15%, 15–30% and 30–100% of the distance from the surface to the geometric center. These ranges were chosen based on prior work indicating that the proliferative normoxic edge typically occupies from 15% to up to 30% of the spheroid radius depending on the spheroid size^21^. The remaining inner region comprises quiescent/hypoxic region and necrotic/anoxic core. For each section, a spheroid mask and Euclidean distance map were generated in Fiji/ImageJ, and the positions of all GFP^+^ pixels were mapped onto the distance image and assigned to the corresponding radial shell (Supplementary Fig. 2). This analysis showed that the fraction of GFP^+^ cells within the outer 0–15% shell was higher in spheroids infected immediately before hanging‑drop formation, whereas the fraction of GFP^+^ cells located in the inner 30–100% region was increased in spheroids infected under ambient oxygen prior to spheroid formation (Fig. 6 C). Together, these data indicate that hypoxia not only decreases adenoviral protein production in 2D culture but also shapes the spatial pattern of adenovirus‑infected cells in 3D spheroids, confining infection largely to the normoxic rim when viral spread occurs parallel with hypoxia development.

## Discussion

The concept of oncolytic virotherapy (OV) dates back over a century, originating from early observations of tumor regression following natural viral infections. The modern use of OV, including adenoviruses, has been under careful investigation for several decades. OV demonstrated clear clinical efficacy, most notably in melanoma patients where talimogene laherparepvec is approved for clinical use^22^. Also, increased therapeutic activity has been observed in patients receiving oncolytic adenoviruses in combination with PD-1/PD-L1 checkpoint inhibitors^23^. However, despite this promise, the use of human adenoviruses in oncolytic therapy still faces several important limitations. One major barrier is hypoxia, a defined feature of solid tumors, which can differentially affect viral infections. While hypoxic conditions can favor certain viruses, for example by contributing to HIV reservoir formation^24^ or enhancing antibody-dependent dengue virus infection^25^, they are detrimental for others. In the case of adenoviruses, multiple studies have shown that hypoxia suppresses viral replication^13,14^. Consistent with these findings, we demonstrate here that hypoxia markedly reduces Hexon production in KP4 cells. Importantly, we further show that hypoxia also shapes adenoviral spread in 3D spheroids, leading to a pronounced spatial restriction of infection to the spheroid periphery.

In virological studies, hypoxic conditions are not always taken into account. Therefore, we first confirmed that cell lines commonly used in adenovirus research - HEK293A and A549 - exhibit the expected cellular responses to hypoxia and to hypoxia-mimicking agents. In addition, we show that the KP4 cell line, although less frequently used in adenoviral studies, displays infection rates comparable to these established models and responds to hypoxia with robust HIF-1α stabilization. A549 and HEK293-derived cell lines are commonly used as CAR-positive reference lines^26–29^. On the other hand, KP4 is a KRAS-mutant pancreatic ductal adenocarcinoma (PDAC) cell line, and CAR expression is likely downregulated^20^, but several alternative mechanisms could still permit adenoviral entry. For example, PDAC cells commonly express αvβ3/αvβ5 integrins^30^, which support penton base-mediated uptake, and show high surface levels of heparan sulfate proteoglycans such as glypican-1^31^ and syndecan-1^32^. KRAS-driven PDAC cells also display strong macropinocytosis^33^. These CAR-independent pathways may explain why KP4 cells are permissive to adenoviral infection.

However, conventional 2D cell cultures cannot recapitulate the complex architecture and microenvironment of solid tumors in vivo. In this context, 3D culture systems, particularly spheroid models, provide a more physiologically relevant platform by capturing spatial organization and dynamic microenvironmental gradients^34,35^. While more complex spheroid systems incorporating stromal components such as fibroblasts, endothelial cells, and immune cells have been developed and are highly valuable for preclinical studies and drug testing, we deliberately focused here on tumor cell-only spheroids. This approach allowed us to specifically address the impact of hypoxia on adenoviral infection.

Several methods are available for spheroid generation. In this study, we chose an approach that does not require additional devices or complex equipment. Therefore, spheroids were generated using the hanging drop technique, which was further optimized by addition of methylcellulose^36^. This approach enabled the reproducible formation of spheroids with comparable initial sizes across all tested cell lines. Nevertheless, differences in spheroid quality were observed. A key requirement for our analysis was sufficient structural stability to withstand cryosectioning. At this stage, only KP4 cells consistently formed spheroids with a stable architecture. Previous studies have shown that spheroids derived from A549 and HEK293 cells can be mechanically fragile and prone to damage during handling^18,37^, and although various spheroid formation techniques have been reported to improve structural stability^38^, optimization of these approaches for our system remains a subject for future investigation. Despite identical seeding densities, KP4 spheroids exhibited the smallest perimeter and area compared with HEK293A and A549 spheroids, indicating a more compact structure. In addition, KP4 spheroids showed remarkably low variability between biological replicates, reflected by minimal standard deviation. Furthermore, KP4 spheroids displayed the highest circularity, suggesting smooth and continuous borders without loose or irregular edges, in contrast to A549 spheroids. They also exhibited the lowest contrast, consistent with a homogeneous cellular distribution throughout the spheroid mass, whereas HEK293A spheroids frequently showed a less dense central region. In addition, as expected, a hypoxic zone was detected within KP4 spheroids using a hypoxia-responsive fluorescent probe.

The impact of hypoxia on viral protein production was evaluated 24 h post exposure. The adenoviral capsid protein Hexon was downregulated under low oxygen compared to controls cultured under ambient oxygen tension. Similar observations have been reported previously, where adenoviral protein levels were reduced under hypoxia, although in those studies different cell lines and different viral proteins, such as E1A, were analyzed^13^. In addition to reduced protein expression, we observed further limitations to adenoviral infection in our 3D spheroid model. Using a GFP-labeled, replication-incompetent adenovirus, we found that infected cells were predominantly localized within the outer ~ 15% of the spheroid radius, corresponding to the normoxic layer, while the hypoxic core remained largely uninfected. GFP expression served as a direct read-out for viral entry and transgene expression. Interestingly, this spatial restriction was markedly reduced when cells were first infected under normoxic conditions and subsequently assembled into spheroids. The precise mechanism underlying the limited GFP detection in the spheroid core remains unclear, but hypoxia may downregulate adenoviral receptor expression^39^ or impair endocytic trafficking^40^, thereby restricting viral uptake. Importantly, such limitations are highly relevant for oncolytic therapy, where efficient delivery of therapeutic genes throughout solid tumors is critical. Comparable spatial constraints have been reported previously using adenoviruses encoding β-galactosidase, where infection was largely restricted to the outer cell layers of spheroids^41^. Notably, future studies should evaluate other HAdV types, including replication-competent vectors, as progeny-mediated dissemination may facilitate improved intraspheroidal distribution.

In conclusion, our study emphasizes the importance of further investigation on the oncolytic adenoviruses, especially utilizing 3D in vitro models that accurately replicate the oxygen gradients present in solid tumors. Using the KP4 cell line, which forms structurally stable spheroids, we demonstrated that it is permissive to infection and can serve as a suitable model to study the spatial distribution of infected cells. Our findings show that hypoxia not only reduces viral protein production in 2D cultures but also limits infection in the hypoxic regions of 3D spheroids. These results underscore the importance of combining both 2D and 3D culture systems to identify optimal adenoviral vectors and advance the development of novel oncolytic virotherapies.

## Materials and methods

### Cell lines and culture conditions

Human A549, HEK293A, and KP4 cells were maintained in DMEM (Thermo Fisher Scientific, Waltham, MA) supplemented with 10% fetal bovine serum and penicillin/streptomycin at 37 °C in a humidified atmosphere containing 5% CO₂. A549 cells were obtained from the European Collection of Authenticated Cell Cultures (ECACC, cat. no. 86012804; Salisbury, UK). HEK293A cells were obtained from Invitrogen (cat. no. R70507; Carlsbad, CA, USA). KP4 cells were kindly provided by working group of Prof. Dr. Jens Siveke, Bridge Institute of Experimental Tumor Therapy, Division of Solid Tumor Translational Oncology and Department of Medical Oncology, West German Cancer Center, University of Duisburg-Essen, University Hospital Essen, Essen, Germany. For hypoxia experiments, cells were cultured at 1% O₂ in a hypoxia workstation (Whitley H35 Hypoxystation, The Don Whitley Scientific Company, Shipley, UK). To stabilize HIF‑1α under normoxia, cells were treated with the prolyl hydroxylase (PHD) inhibitors Roxadustat (20 or 40 µM; Cayman Chemical, Hamburg, Germany) or Dimethyloxalylglycine (DMOG; 125 or 250 µM; Cayman Chemical, Hamburg, Germany) for the indicated durations. DMSO served as vehicle control. For 2D infection experiment, KP4 cells were incubated with HAdV5 at the indicated MOI under ambient oxygen conditions for 2 h. Following this, cells were washed and cultured either under ambient oxygen or 1% O₂ for 24 h prior to protein extraction and subsequent analyses.

### Hanging drop method for spheroid formation

Spheroids were generated using a hanging‑drop technique. A cell suspension was prepared in DMEM supplemented with 10% FCS, 1% penicillin/streptomycin and 0.24% (w/v) methylcellulose (Merck KGaA, Darmstadt, Germany). For experiments involving magnetic stirring, cells were initially cultured for 24 h in DMEM supplemented with 10% FCS and 1% penicillin/streptomycin under stirring conditions, after which the medium was replaced with methylcellulose-containing one. For experiments requiring visualization of hypoxic regions, the suspension was additionally supplemented with Image-iT™ Green Hypoxia reagent according to the manufacturer’s instructions (Thermo Fisher Scientific, Waltham, MA, USA). Droplets of 50 µl containing 50,000 cells were carefully pipetted onto the inner surface of 100‑mm dish lids and the lids were inverted over dishes containing 10 ml PBS to create a humid chamber. The hanging‑drop cultures were incubated at 37 °C in 5% CO₂ for 5 days. To harvest spheroids, PBS was gently pipetted onto the lid to detach the aggregates, which were then collected using a sterile pipette tip with a widened opening and transferred into microcentrifuge tubes. Spheroids were fixed in 4% paraformaldehyde for 2 h on a rocking platform at room temperature and washed twice with PBS. When indicated, spheroids were permeabilized with 0,1% Triton X‑100 and stained with DAPI for 2 h before further analysis.

### Virus

Wild-type HAdV5 and the E1-/E3-deleted HAdV5_GFP encoding green fluorescent protein (GFP) under the control of the human CMV IE1 promoter were used in this study. Virus cultivation, purification, and titration were performed as previously described^15,42^.

### Histological evaluation

Fixed spheroids were embedded in Tissue‑Tek OCT compound (Sakura Finetek, Tokyo, Japan) and sectioned using standard procedures. When cryosectioning was performed, sections were prepared with the aid of Kawamoto’s adhesive film (Section Lab Co. Ltd., Yokohama, Japan) to preserve spheroid morphology. Sections were mounted on glass slides and either left unstained to visualize HAdV5_GFP signal or Hypoxia Green dye, or stained with hematoxylin and eosin for general histological assessment.

### Microscopy

Bright‑field images of intact spheroids were acquired using a DMi1 microscope (Leica Microsystems Wetzlar, Germany). Fluorescence images of whole spheroids and spheroid sections were obtained on a Nikon Eclipse Ts2 microscope (Nikon Instruments, Tokyo, Japan) or a Zeiss Axiovert 200 M microscope (Carl Zeiss, Oberkochen, Germany) and analyzed using NIS‑Elements (Nikon) or Imaris (Oxford Instruments, Abingdon, UK). Z‑stack image series were collected on the Zeiss Axiovert 200 M microscope and processed in Imaris. Quantitative analysis of spheroid morphology was performed using the open-source Insidia macro implemented on the Fiji/ImageJ image-analysis platform^43^. The parameters extracted included spheroid perimeter (in pixels), spheroid area (in pixels²), circularity and contrast. Circularity was calculated as 4π*area/perimeter^2^, where a value of 1.0 indicates a perfect circle and values approaching 0.0 indicate increasingly elongated shapes. Contrast, reflecting image heterogeneity, was used as a measure of gray-level variation and spatial disorder within the spheroid image.

### Quantification of spatial distribution of infected cells in spheroids

Fluorescence microscopy images (GFP channel) and corresponding brightfield images of spheroid cryosections were analyzed using Fiji/ImageJ (NIH). The spheroid outline was manually segmented from the brightfield image to generate a binary mask defining the spheroid area. Artifacts and background noise were removed using thresholding followed by morphological cleaning steps. The binary mask was used to generate a distance map (Euclidean Distance Map), where each pixel value represented the shortest distance to the spheroid surface. GFP-positive signals were segmented from the fluorescence channel using intensity thresholding to create a GFP mask, which was restricted to the spheroid area using the spheroid mask. Individual GFP^+^ regions were identified using particle analysis, and regions of interest (ROIs) were transferred to the distance map image to extract the mean distance value for each GFP^+^ region. To enable normalization between images, distance values were normalized to the global maximum distance of the corresponding distance map (representing the deepest interior point of each spheroid section), resulting in relative distance values ranging from 0 (surface) to 1 (core). Normalized distances were used for subsequent quantitative analysis of the spatial localization of infected cells within spheroids. Calculations were performed from 2 to 4 sections of 3 spheroids per condition.

### Protein isolation and Western blot

Cells were lysed in a buffer containing 150 mM NaCl, 20 mM Tris (pH 7.5), 5 mM EDTA, and 1% Nonidet P-40, supplemented with 10% freshly added protease inhibitor (Roche Diagnostics, Basel, Switzerland), and incubated on wet ice for 30 min. Lysates were centrifuged at 10,000×g for 5 min at 4 °C, and the resulting supernatant was collected and stored at − 80 °C until further analysis. Protein concentrations were quantified using the Bio-Rad protein assay reagent (Bio-Rad, Munich, Germany) and values were referenced to a bovine serum albumin standard. Equal amounts of protein (30 µg per sample) were denatured in sodium dodecyl sulfate (SDS) buffer and separated by SDS–polyacrylamide gel electrophoresis. Proteins were transferred onto polyvinylidene difluoride membranes (peqlab, VWR International, Darmstadt, Germany) using the Trans-Blot^®^ Turbo™ Transfer System (Bio-Rad, Munich, Germany). Membranes were blocked with 5% milk in Tris-buffered saline containing 0.05% Tween and incubated with primary antibodies against α-tubulin (Santa Cruz Biotechnology, Dallas, TX, USA)^44^, HIF-1α (BD Transduction Laboratories™, Franklin Lakes, NJ, USA)^45^, β-actin (Sigma-Aldrich, St. Louis, MO, USA)^45^ and adenovirus Hexon (Novus Biologicals, Centennial, CO, USA)^46^, diluted in the same blocking solution. Following incubation with horseradish peroxidase-conjugated secondary antibodies (anti-rabbit or anti-mouse), chemiluminescence detection was performed using the SignalBright Max Protein Detection Kit (Proteintech, Rosemont, IL, USA), and signals were visualized with the Fusion FX imaging system (Vilber Smart Signaling, Marne-la-Vallée Cedex 3, France).

### Flow cytometry

Cells were washed and transferred into a 96-well plate, followed by staining with a fixable viability dye (Thermo Fisher Scientific, Waltham, MA, USA) to enable exclusion of dead cells during subsequent analysis. The viability staining was performed for 20 min at room temperature in the dark. When it was necessary, cells were fixed for 2 h afterwards using the eBioscience Foxp3/Transcription Factor Staining Buffer Set (Thermo Fisher Scientific, Waltham, MA, USA) and subsequently incubated overnight with antibodies against HIF-1α (Clone 54/HIF-1α, BD Biosciences, San Jose, CA, USA) for intracellular staining. Samples were acquired on a FACSCelesta flow cytometer (BD Biosciences, San Jose, CA, USA), and data were analyzed with FlowJo software (BD Biosciences, San Jose, CA, USA).

## Supplementary Information

Below is the link to the electronic supplementary material.

Supplementary Material 1

Supplementary Material 2

Supplementary Material 3

Supplementary Material 4

## Data Availability

The data generated in this study are publicly available in the Sciebo repository at: https:/uni-duisburg-essen.sciebo.de/s/yYjSDckRFfniFQw.
