# Supplementary material for: Spheroids reveal hypoxia‑driven spatial restriction of adenoviral infection
Source: Sci Rep. 2026 May 21;16:15864. doi: 10.1038/s41598-026-53319-4 (PMC13194710; doi:10.1038/s41598-026-53319-4)
Supplement: Supplementary file 3 — Supplementary Material 3 [file 41598_2026_53319_MOESM3_ESM.pdf]

Supplementary Figure 1. Original uncropped images of the Western Blot assays

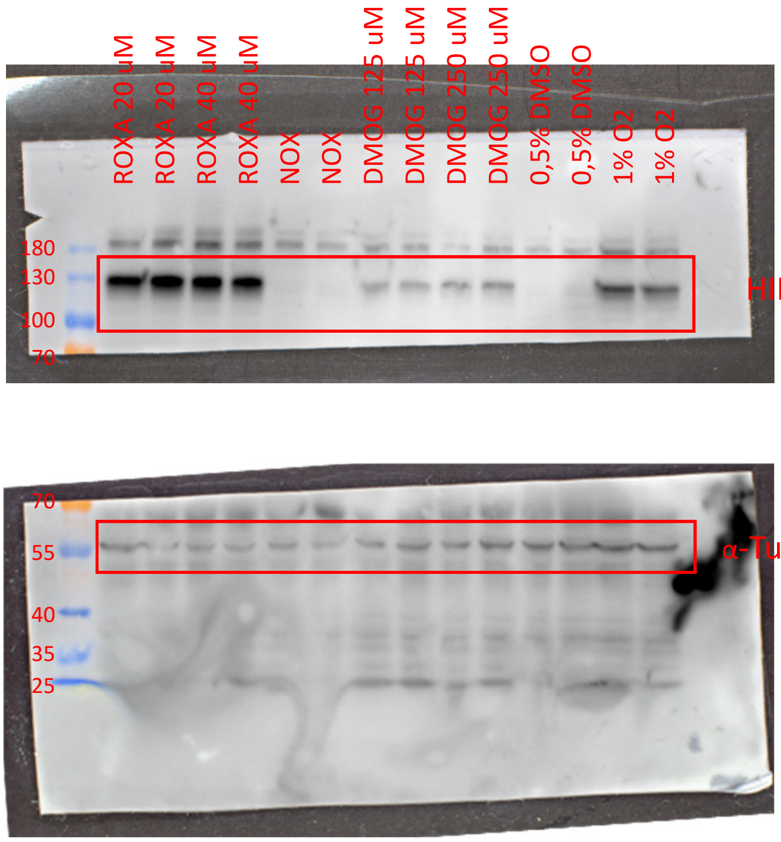

HEK293A

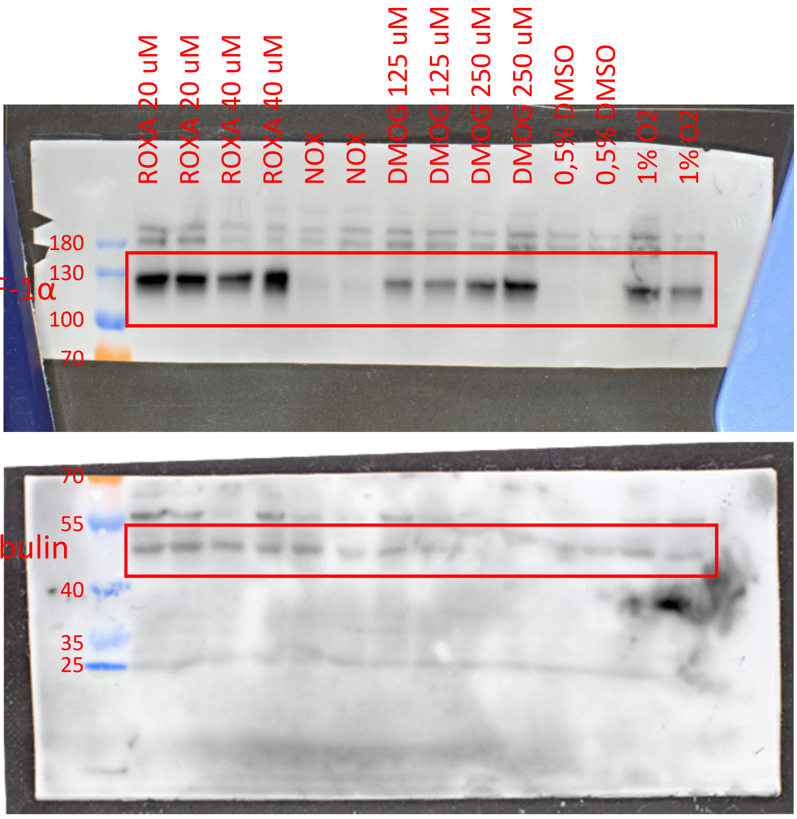

A549

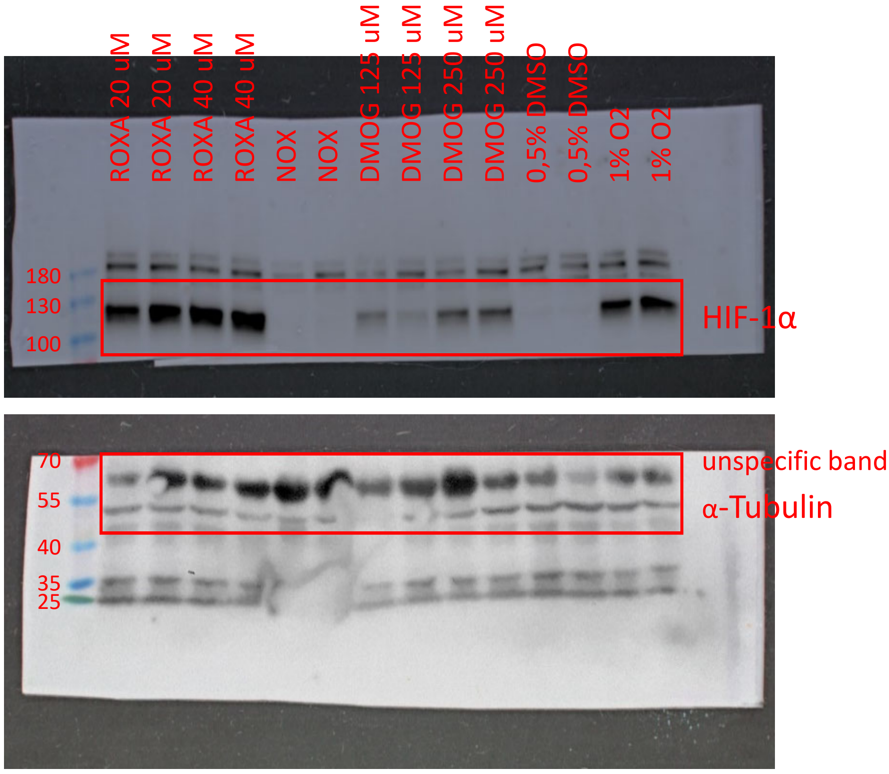

KP4

Original uncropped images of the Western blots presented in Figure 1. The regions of the cropped blots have been denoted using red boxes.

Supplementary Figure 1. Original uncropped images of the Western Blot assays

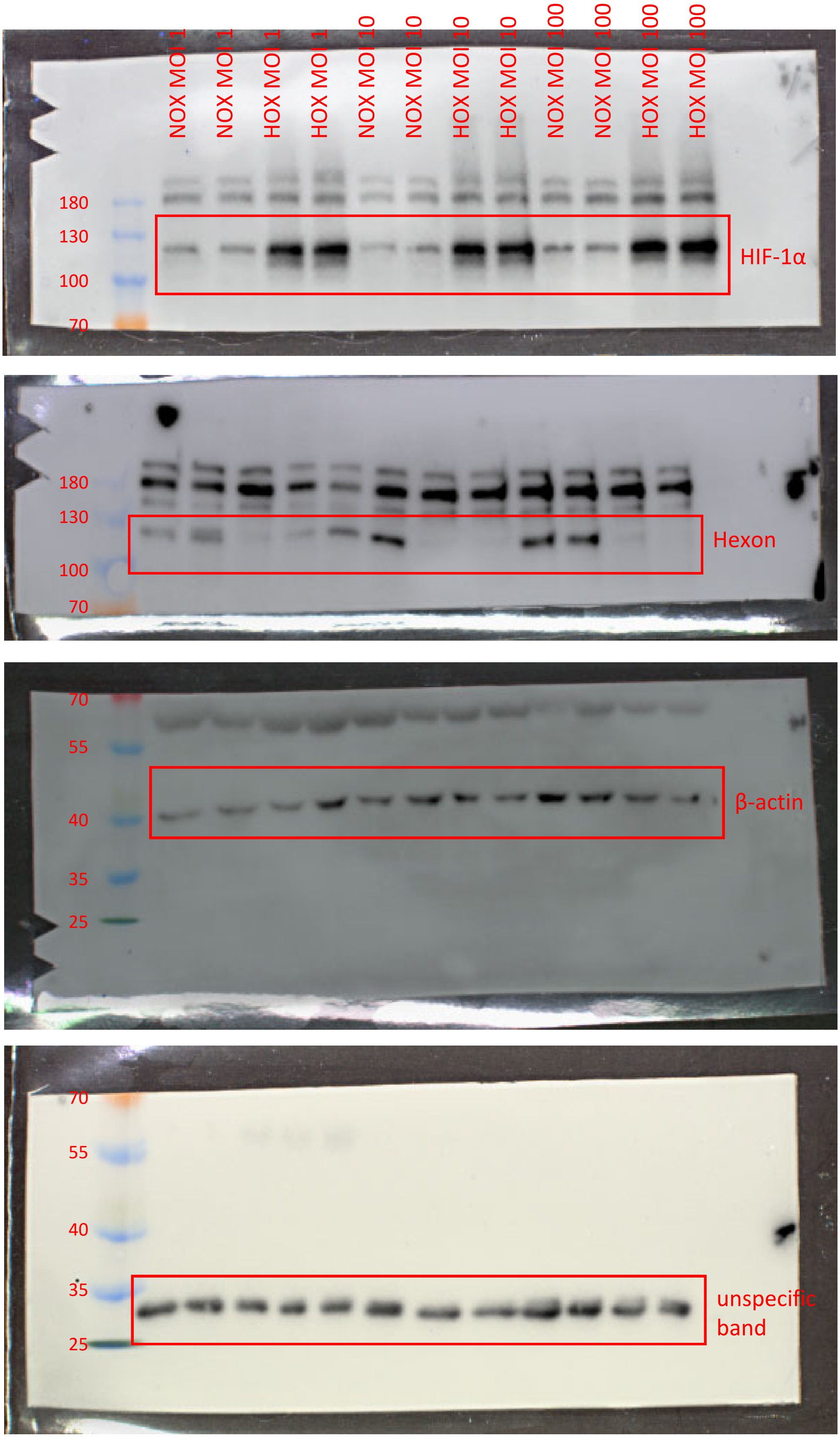

Original uncropped images of the Western blots presented in Figure 4. The regions of the cropped blots have been denoted using red boxes.
