# Supplementary material for: Spheroids reveal hypoxia‑driven spatial restriction of adenoviral infection
Source: Sci Rep. 2026 May 21;16:15864. doi: 10.1038/s41598-026-53319-4 (PMC13194710; doi:10.1038/s41598-026-53319-4)
Supplement: Supplementary file 4 — Supplementary Material 4 [file 41598_2026_53319_MOESM4_ESM.pdf]

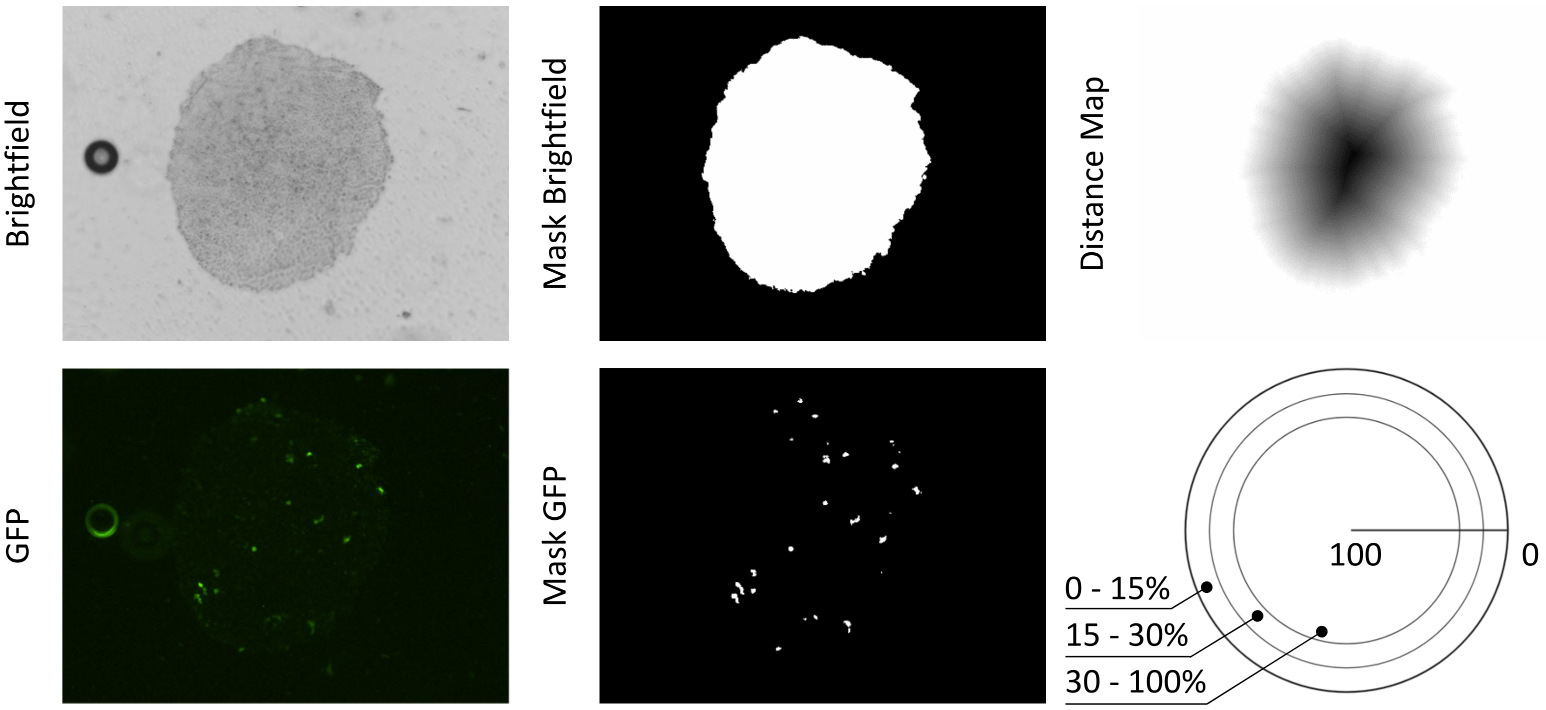

Supplementary Figure 2. Workflow for quantifying the distance of HAdV5_GFP^{+} cells from the spheroid surface. Images show a representative section of a KP4 spheroid infected with HAdV5_GFP at MOI 1000, agitated for 24 h before spheroid formation, and fixed on day 6 post infection. Brightfield images of spheroid sections were first used to generate a binary spheroid mask by intensity thresholding (top row). The spheroid mask was then converted into a Euclidean distance map, in which each pixel value represents the distance from the spheroid boundary. In parallel, the GFP channel was thresholded to obtain a binary mask of HAdV5_GFP-positive regions (bottom row). Distances of GFP-positive pixels to the spheroid surface were extracted by multiplying the GFP mask with the distance map, normalized and grouped into concentric shells (0 - 15%, 15 - 30%, 30 - 100% of spheroid radius, where 0% represents surface, 100% represents core).
